# Socioeconomic status and the growth of intelligence from infancy through adolescence

**DOI:** 10.1016/j.intell.2014.10.002

**Published:** 2015

**Authors:** Sophie von Stumm, Robert Plomin

**Affiliations:** a Department of Psychology, Goldsmiths University of London, SE14 6NW London, UK; b MRC Social, Genetic and Developmental Psychiatry Centre, King's College London, Institute of Psychiatry PO80, De Crespigny Park, SE5 8AF London, UK

**Keywords:** Intelligence, IQ, Socioeconomic status, Latent growth, Gender

## Abstract

Low socioeconomic status (SES) children perform on average worse on intelligence tests than children from higher SES backgrounds, but the developmental relationship between intelligence and SES has not been adequately investigated. Here, we use latent growth curve (LGC) models to assess associations between SES and individual differences in the intelligence starting point (intercept) and in the rate and direction of change in scores (slope and quadratic term) from infancy through adolescence in 14,853 children from the Twins Early Development Study (TEDS), assessed 9 times on IQ between the ages of 2 and 16 years. SES was significantly associated with intelligence growth factors: higher SES was related both to a higher starting point in infancy and to greater gains in intelligence over time. Specifically, children from low SES families scored on average 6 IQ points lower at age 2 than children from high SES backgrounds; by age 16, this difference had almost tripled. Although these key results did not vary across girls and boys, we observed gender differences in the development of intelligence in early childhood. Overall, SES was shown to be associated with individual differences in intercepts as well as slopes of intelligence. However, this finding does not warrant causal interpretations of the relationship between SES and the development of intelligence.

## Highlights

• IQ growth trajectories were modeled in British children from age 2 to 16 years.
• Children's socioeconomic background (SES) was associated with IQ growth.
• High and low SES children differed by 6 IQ points at age 2.
• By age 16, this IQ difference between high and low SES children had tripled.

## 1 Introduction

Individual differences in intelligence influence developmental trajectories across the lifespan, affecting socioeconomic, psychological, and health outcomes (Deary, 2012). Differences in intelligence have been shown to be highly stable from early adolescence to late adulthood (Deary, Pattie, & Starr, 2013), but are more variable in infancy and childhood, with some children showing substantial gains in intelligence and others considerable losses between infancy and adolescence (Bayley, 1955, Feinstein, 2003, Tucker-Drob and Briley, 2014). These variations in the development of intelligence are likely to be associated with children's family socioeconomic status (SES; e.g. Dyume et al., 1999, Heckman, 2006, Tucker-Drob et al., 2011). Children from disadvantaged family backgrounds score on average lower on intelligence tests than their high SES peers (Bradley and Corwyn, 2002, Schoon et al., 2012, Strenze, 2007), and their performance has been suggested to worsen over time, even if they did relatively well in early assessments (Feinstein, 2003). Conversely, high SES children are thought to gain in intelligence over time, even if they initially had a lower test score (Feinstein, 2003). However, research to date on the impact of SES on developmental change in intelligence is inconclusive for two reasons.

First, it has been suggested that the previously reported association between SES and children's IQ development results from regression to the mean, because children with either extremely high or low scores in early IQ tests are less likely to score as extremely in later tests, independent of their family background (Jerrim and Vignoles, 2011, Saunders, 2012). Regression to the mean occurs when children are grouped according to their scores at one measurement occasion and then the groups' development is analyzed across subsequent assessments. This statistical artifact can be avoided by applying latent growth curve (LGC) models to non-selected samples, because LGC analyzes longitudinal data at the level of individuals rather than groups.

Second, most previous studies included intelligence assessments at relatively few ages and at short age intervals in early life (Feinstein, 2003, Spinath et al., 2003, von Stumm, 2012; see Tucker-Drob & Briley, 2014, for a review). No study to date has modeled change in intelligence in a representative sample from infancy through late adolescence, using multiple assessments of intelligence over time that allow for identifying individual differences in developmental trajectories. Overcoming these limitations, in the present study we fitted LGC models to intelligence data from the Twins Early Development Study (TEDS), whose participants were assessed 9 times on intelligence from age 2 to 16 years. We then tested the extent to which SES, as a time-invariant covariate, accounted for individual differences in slopes of change in intelligence from age 2 to 16 years, as well as individual differences in the starting points (intercepts) at the age of 2 years.

## 2 Methods

### 2.1 Sample

The Twins Early Development Study (TEDS) recruited families of twins born in England and Wales in 1994, 1995, and 1996 (Haworth, Davis, & Plomin, 2013). Since then, the sample has remained representative of the UK population (Kovas, Haworth, Dale, & Plomin, 2007). We excluded twins who suffered from severe medical problems currently or at birth (e.g., post-natal surgery); whose mothers reported severe medical problems during pregnancy; whose first language was not English; and who had been assessed less than twice on intelligence between the ages of 2 and 16 years. The final analysis sample comprised of 14,853 twins; that is, 7426 complete pairs, including 2564 monozygotic pairs and 4862 dizygotic twin pairs, of which 2375 were of opposite sex. Overall, the sample included 7768 girls and 7085 boys.

### 2.2 Measures

#### 2.2.1 Socioeconomic status (SES)

Parental education and occupation (mother's and father's highest educational qualification and job status) were recorded at the first contact with the families, when twins were 18 months old, and again when they were 7 years old. Family income was assessed when the twins were 9 years old. A composite of parental education and occupation at twins' age of 18 months correlated at .77 with a composite of parental education and occupation at twins' age of 7 years, which in turn correlated at .57 with family income at twins' age of 9 years, suggesting that SES was relatively stable over time in TEDS (Hanscombe et al., 2012). Data from the assessment at 18 months were used in cases where information at age 7 was missing; for all others, records of parental education and occupation at age 7 were employed. Summary SES composites were created as a unit-weighted sum of the education, occupation, and income after mapping to a standard normal distribution with the rank-based van der Waerden transformation (Hanscombe et al., 2012).

#### 2.2.2 Intelligence

The twins were assessed at 2, 3, 4, 7, 9, 10, 12, 14 and 16 years on intelligence, using parent-administered tests and ratings of ability at ages 2, 3, and 4, and a mixture of web-based, telephone-based, and parent-administered tests at later ages. At each testing age, twins completed at least two state-of-the-art ability tests. The tests have been described in detail elsewhere (Hanscombe et al., 2012) and are only briefly reviewed here.

##### 2.2.2.1 Measures at ages 2, 3, and 4

Parent-administered tests and parent-reported observations were used to assess verbal and nonverbal cognitive abilities. These measures have been validated against standard tests administered by trained testers (Oliver et al., 2002, Saudino et al., 1998). Specifically, nonverbal cognitive performance was assessed using age-appropriate versions of the Parent Report of Children's Abilities (PARCA; Oliver et al., 2002, Saudino et al., 1998), while verbal ability measures included vocabulary and grammar as assessed by parent reports for the CDI–III, an extension of the short form of the MacArthur Communicative Development Inventories: Words and Sentences (Fenson et al., 2000).

##### 2.2.2.2 Measures at age 7

Children were tested on verbal and nonverbal abilities by telephone (Petrill, Rempell, Oliver, & Plomin, 2002). Prior to the telephone call, parents were sent a booklet of test items along with testing instructions. The booklet contained two tests of verbal tests: the Similarities subtest and the Vocabulary subtest from the Wechsler Intelligence Scale for Children (WISC-III-UK; Wechsler, 1992), and the Picture Completion subtest from the WISC-III-UK and Conceptual Grouping from the McCarthy Scales of Children's Abilities (McCarthy, 1972).

##### 2.2.2.3 Measures at age 9

Participants were mailed a test booklet with two verbal and two nonverbal tests to be administered under the supervision of the parent, who had received a corresponding instruction booklet. The verbal tests comprised vocabulary and general knowledge tests adapted from the multiple-choice version of the WISC-III-UK (Wechsler, 1992). The nonverbal tests included a Puzzle test adapted from the Figure Classification subtest of the Cognitive Abilities Test 3 (CAT3; Smith, Fernandes, & Strand, 2001) and a Shapes test also adapted from the CAT3 Figure Analogies subtest (Davis, Arden, & Plomin, 2008).

##### 2.2.2.4 Measures at age 10

Testing was web-based, and children completed two verbal and two non-verbal tests using their home computers (Haworth et al., 2007). Tests were drawn from the WISC-III-PI, including Multiple Choice Information (General Knowledge), Vocabulary Multiple Choice, and Picture Completion (Wechsler, 1992), and from Raven's Standard Progressive Matrices (Raven, Court, & Raven, 1996).

##### 2.2.2.5 Measures at age 12

Testing was web-based and conducted at home computers, using age-matched versions of the tests previously used at age 10, including again two verbal and two non-verbal ability tests (Kaplan et al., 1998, Raven et al., 1996, Wechsler, 1992).

##### 2.2.2.6 Measures at age 14

Twins completed two web-based tests at their home computers, including the WISC-III-PI Vocabulary Multiple Choice for 14-year olds (Kaplan et al., 1998), and Raven's Progressive Matrices (Raven et al., 1996).

##### 2.2.2.7 Measures at age 16

Twins completed web-based adaptations of Raven's Standard and Advanced Progressive and the Mill-Hill Vocabulary Scale using their home computers (Raven et al., 1998, Raven et al., 1996).

## 3 Analysis

### 3.1 Latent growth curve models

In a first step, first principal factors were extracted at each age from the administered intelligence tests. Regression factor scores were transformed into standardized IQ scores with a mean of 100 and a standard deviation of 15 (Hanscombe et al., 2012). The comparability of IQ scores is here theoretically inferred, because intelligence was assessed at each measurement occasion with multiple, well validated tests that should have assessed identical constructs, yielding invariant common variance factors of intelligence, even if different tests were administered at different times and ages. Previously, multiple first factors extracted from cognitive test batteries were shown to be invariant in adults (Johnson et al., 2004, Johnson et al., 2008), although the invariance of such factors in children or over the course of time has not been established.

In a second step, latent growth curve (LGC) models were fitted to two subsamples that each consisted of one twin randomly selected from a pair (sample 1 with N = 7440, and sample 2 with N = 7413). This method enabled a replication of the LGC model across the two samples, and it also ensured that model fit statistics were not erroneously inflated because of the dependence of observations (i.e. relatedness of twins). LGC factors are extracted from repeatedly observed intelligence factor scores and describe a sample's average starting point, typically referred to as intercept, and systematic changes that occur over time, which are typically known as slope (McArdle, 2009). LGC factors were modeled to freely correlate. To determine the number of LGC factors that best represented the data, the fit (i.e. χ^2^(df)) of a LGC model with two growth parameters (intercept and slope) was compared to the fit of a model with three growth parameters (intercept, slope and quadratic term). That is, the slope represents linear changes, while the quadratic growth parameter assesses systematic non-linear accelerations or decelerations of the growth trend (i.e. systematic curvilinear change not accounted for by the slope). At each age, loadings on the intercept were fixed to 1, and those on the slope were defined as 0, 1, 2, 5, 7, 8, 10, 12, and 14, representing *time periods* in years between each assessment point, ranging in *real time* from 2 to 16 years. With that, the intercept was defined where the slope had a zero loading (i.e. at age 2). Loadings on the quadratic term were the square of the slope loadings. Both 2- and 3-factor LGC models were specified as multi-group models to test for measurement invariance across the two random twin subsamples. Here, the fit of an ‘unrestricted’ baseline multi-group LGC model was compared to a restricted model that held means, intercepts and residuals equal across groups.^1^

Next, multi-group LGC models were fitted separately to samples of boys and girls investigate if LGC factors differed across gender. Accordingly, the fit of a baseline model, which was ‘unrestricted’ besides pre-defined factor loadings^1^, was compared to the fit of a ‘restricted’ model that held intercepts, means and residuals equal across boys and girls. Finally, SES was added to the LGC model and specified as a time-invariant covariate of the growth parameters in order to investigate the extent to which LGC factors differed as a function of SES.

All models were fitted using full information maximum likelihood estimation (FIML) assuming data missing at random and no biases of the results (Arbuckle, 1996). Several fit indices evaluated the LGC models' fit, including the model χ^2^ test, the Comparative Fit Index (CFI), the Tucker–Lewis Index (TLI), and the RMSEA with Confidence Intervals of 95% (Hu & Bentler, 1999).

## 4 Results

### 4.1 Correlations

Across ages, intelligence scores were positively inter-correlated in a simplex pattern, with stronger associations between more proximate assessments, in line with previous studies of different samples (e.g., Bartels, Rietveld, van Baal, & Boomsma, 2002; Table 1). SES correlated positively with intelligence at all ages, and increasingly so, as the children grew older, which is also in line with previous research (e.g. Tucker-Drob et al., 2011).

The correlations of intelligence scores over time in TEDS ranged from .21 to .70 with an average value of .40, which may appear low compared to other research (e.g. Deary et al., 2013). However, intelligence is more variable in childhood than in later life (e.g. Bayley, 1955); also, intelligence assessment methods in TEDS varied considerably across time (see Discussion for details).

### 4.2 Latent growth curve models

A two-factor latent growth curve model fitted the data worse (sample 1 with N = 7440: χ^2^ (40) = 1090.35; sample 2 with N = 7413: χ^2^ (40) = 1141.62) than the three-factor model in both twin subsamples (sample 1: χ^2^ (36) = 553.67; sample 2: χ^2^ (36) = 602.31). In multi-group models across two samples of one twin randomly selected from a pair, LGC model χ^2^ values did not differ significantly. Thus, individual differences in intelligence from age 2 to 16 years were here best explained by an intercept (average starting point), and factors of linear (slope) and non-linear (quadratic term) change. All three growth factors accounted for significant variance, suggesting individual differences in intercept and in linear and non-linear change.

**Fig. 1:**
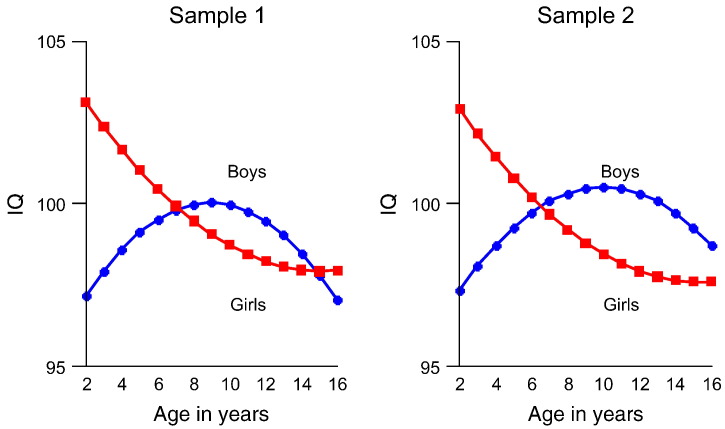
Latent IQ growth curves for boys and girls from age 2 to 16 years in two subsamples of one randomly selected twin per pair from TEDS. *Note.* Gender differences in latent growth curves were significant. Models did not differ significantly between subsamples 1 and 2, confirming the measurement invariance of the LGC model across samples of twin siblings.

In the multi-group models for gender, the fit of unrestricted models differed significantly from the fit of models that held intercepts, means and residuals equal (*p* < .001; Table 2). In other words, boys and girls differed in their developmental trajectories of intelligence: girls started with an advantage of about 5 IQ points at the age of 2 years compared to boys. However due to different values for slope and quadratic term in boys and girls (Table 2), the gender difference in IQ development had mostly disappeared by the age of 16 years (Fig. 1). Specifically, the slope was negative in girls, while the quadratic term was positive. As a result, girls had on average a higher IQ starting point but showed decline thereafter, which was somewhat absorbed by the quadratic term. By comparison, boys' slope was positive and the quadratic negative: thus, they improved from their low average IQ starting point but the negative quadratic term dampened and even reversed the IQ growth trend over time.

### 4.3 Associations between SES and latent growth curve factors

SES was a significant predictor of all three latent growth factors in boys and girls across two subsamples of twins (*p* < .001 in all cases, Table 3). The χ^2^ of a model that held means, intercepts, and residuals equal across groups did not differ significantly from the χ^2^ associated with a model that also constrained the SES regression parameters to be equal across groups (*p* > .05 in all cases). Thus, the association between SES and intelligence latent growth factors did not vary as a function of gender or twin subsample.

**Fig. 2:**
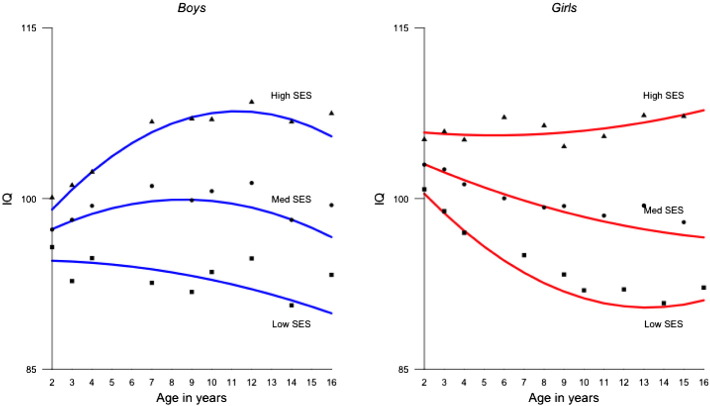
IQ growth curves according to SES background for boys and girls in TEDS. *Note.* Lines refer to latent growth curve trajectories. Dots represent the IQ raw means. High SES (triangles) refers to children, whose family SES was at least 1 SD above the SES mean; low SES (squares) refers to children from families who scored 1 SD below the SES mean. Medium SES (dots) includes all children, whose families were between − 1 and + 1 SD of SES.

SES was positively associated with the intercept for intelligence with coefficients ranging from .11 to .15, suggesting that children from more advantaged SES backgrounds had higher intelligence scores in infancy. SES was also positively related to the slope with coefficients ranging from .20 to .26, which indicates that children from more advantaged SES backgrounds also experienced greater linear gains from age 2 to 16 years. Associations between SES and the quadratic term were negative, with coefficients ranging from − .21 to − .15, which in combination with the observed gender differences in latent growth factors resulted in differently shaped growth curves. In boys, the negative association of SES with the negative quadratic term implied a greater steepness of the growth curves as SES increased. Conversely in girls, the negative association between SES and a positive quadratic term resulted in flatter growth curves with higher SES. Fig. 2 illustrates the relationship between SES and latent growth in intelligence for boys and girls from low (<− 1 SD), medium (±1 SD) and high (> 1 SD) SES families. Both boys and girls from low SES backgrounds scored on average about 6 IQ points lower at age 2 than children from high SES family backgrounds. By age 16, this discrepancy had multiplied: low SES boys scored on average 15 IQ points less than high SES boys, and in girls this difference amounted to approximately 17 IQ points.

## 5 Discussion

Our results suggest that family socioeconomic status (SES) impacts children's development of intelligence from infancy through adolescence. Children of the highest and lowest SES backgrounds were on average separated by 6 IQ points at the age of 2 years. By the age of 16, the IQ gap had almost tripled (Fig. 2). Thus, children from more disadvantaged families not only did worse than their peers in early intelligence tests but their intelligence handicap amplified over time, suggesting a long-term agglomeration of SES influences on cognitive development. We want to emphasize here that these SES influences comprise not only environmental variance but also their association with children's cognitive growth is likely to be partially mediated by genetic factors (Trzaskowski et al., 2014). However, the current study design only allows for speculating about the mechanisms that potentially underlie the association between SES and IQ growth. It is plausible that children from higher SES families experience greater opportunities for and support in cognitive engagement and learning than children from more disadvantaged homes (Bradley & Corwyn, 2002). Differences in the availability of learning opportunities, support and resources are thought to accentuate individual differences in cognitive ability (Hayes, 1962, von Stumm, 2012). That said, the precise mechanisms underlying the association between SES and intelligence growth curves are yet to be identified.

We also observed significant gender differences in the IQ starting point and in the growth curves for cognitive development, with girls outperforming boys at the ages of 2, 3, and 4 years. However in later childhood and adolescence, gender differences in cognitive growth diminished and by the age of 16 years, the differences had disappeared. Our findings concur with reports about gender differences in cognitive abilities and in brain anatomy (e.g. Ganjavi et al., 2011, Haier et al., 2005). However to our knowledge, no comparable longitudinal data are available that would allow replicating the pattern of gender differences in cognitive development that we observed here. Furthermore, we found no differences in the association between SES and cognitive development across boys and girls.

### 5.1 Strengths and limitations

Our study has many notable strengths, including a large sample of twins representative of the UK population (Kovas et al., 2007) with intelligence assessed 9 times between the ages of 2 and 16 years. The main weakness for longitudinal comparisons is that different measures of and assessment methods for intelligence were used as different ages, thus confounding age and methodological differences. For the initial three assessment waves at the twins' ages of 2, 3, and 4 years, tests were parent-administered, but at later ages, children completed phone-based and web-based IQ tests without much parental involvement. These measurement differences may have resulted in our lower than expected correlations between intelligence scores across twins' ages. That said, the correlations observed here are only marginally lower compared to estimates from other samples at comparable ages (Bayley, 1955, Bartels et al., 2002, Lobo and Galloway, 2013). A second limitation is our treatment of family SES as a time-invariant covariate in the analyses, although SES indicators did vary over time in our sample (Hanscombe et al., 2012). Notwithstanding, the stability of SES was here greater than its degree of change with correlations of three SES measurements exceeding .5 over a period of 7 years, suggesting that treating SES as time-invariant covariate was appropriate. A third limitation is that, although our sample consisted of twins, we did not conduct genetic analyses, primarily because SES is a between-family variable. As such, it is not amenable to genetic analysis using the twin method, which relies on within family differences.

## 6 Conclusions

This study showed that children from lower SES backgrounds tend to perform on average worse on intelligence tests than children from more privileged homes as early as at the age of 2 years. Furthermore, SES accentuated these differences throughout childhood and adolescence: the 6-point IQ difference in infancy between children from low and high SES homes almost tripled by the time the children were 16 years old. Our findings confirm changes in intelligence throughout early life and suggest a meaningful relationship between IQ growth and socioeconomic factors.

## Figures and Tables

**Table 1:** Sample sizes and correlations for the IQ and SES data in TEDS from age 2 to 16 years for a subsample of one randomly selected twin per pair.

		N	1	2	3	4	5	6	7	8	9
1	IQ at 2	4730	–								
2	IQ at 3	4522	.66	–							
3	IQ at 4	5725	.57	.70	–						
4	IQ at 7	4620	.23	.31	.31	–					
5	IQ at 9	3059	.26	.35	.33	.41	–				
6	IQ at 10	2475	.23	.31	.27	.40	.57	–			
7	IQ at 12	3981	.18	.27	.29	.44	.56	.63	–		
8	IQ at 14	2599	.21	.26	.24	.40	.46	.51	.63	–	
9	IQ at 16	2224	.21	.26	.22	.42	.45	.50	.58	.64	–
10	SES	6884	.10	.17	.17	.32	.30	.26	.30	.36	.35

*Note.* Correlations were computed after pairwise deletion in a subsample of one randomly selected twin per pair.

**Table 2:** Sample sizes, model χ^2^, and latent growth factor parameters in boys and girls across two subsamples, each of one twin randomly selected from a pair, in TEDS.

	Boys 1	Girls 1	Boys 2	Girls 2
N	3549	3891	3536	3877
χ^2^(36)	361.27	298.94	402.45	285.94
Intercept	97.17	103.13	97.33	102.93
SE_I_	0.27	0.26	0.27	0.25
Variance_I_	163.52	162.21	164.25	159.42
CI (95%)_I_	96.64 to 97.70	102.63 to 103.63	96.80 to 97.86	102.43 to 103.42
Slope	0.83	− 0.79	0.80	− 0.80
SE_S_	0.09	0.08	0.09	0.08
Variance_S_	7.44	7.55	8.06	7.25
CI (95%)_S_	0.66 to 0.99	− 0.94 to − 0.64	0.64 to 0.97	− 0.95 to − 0.64
Quadratic	− 0.06	0.03	− 0.05	0.03
SE_Q_	0.01	0.01	0.01	0.01
Variance_Q_	0.02	0.02	0.03	0.02
CI (95%)_Q_	− 0.07 to − 0.04	0.02 to 0.05	− 0.06 to − 0.04	0.02 to 0.04

*Note.* Fit indices for the multi-group model in sample 1 were: CFI = .947; TLI .947; and RMSEA = .047 (CI 95% .044 to .050). Fit indices for the multi-group model in sample 2 were: CFI = .944; TLI = .944; and RMSEA = .048 (CI 95% .045 to .051). SE refers to Standard Error; CI 95% refers to Confidence Interval of 95%; the subscripts I, S, and Q refer to intercept, slope and quadratic term respectively.

**Table 3:** Regression parameters for the association between SES and IQ latent growth factors in boys and girls across two subsamples of twins from TEDS.

	B_i_	SE_i_	β_i_	B_s_	SE_s_	β_s_	B_q_	SE_q_	β_q_
Boys 1	2.19	.39	.12	0.98	.12	.26	− 0.05	.01	− .21
Girls 1	2.73	.37	.15	0.81	.11	.21	− 0.03	.01	− .16
Boys 2	2.05	.39	.11	0.95	.12	.24	− 0.04	.01	− .19
Girls 2	2.70	.37	.15	0.75	.11	.20	− 0.03	.01	− .15

*Note.* B refers to the unstandardized regression estimate; SE is the Standard Error; and β is the standardized regression coefficient. Subscripts denote the latent growth factor that values refer to (i = intercept; s = slope; q = quadratic term). All regression coefficients are significant at *p* < .001.

## Footnotes

1 Configural measurement invariance is given in LGC multi-group models, because factor loadings are pre-defined to describe intercept (loadings of 1), slope (loadings from 0 to 14) and quadratic term (loadings from 0 to 196).
